# Multi-Resolution Learning and Semantic Edge Enhancement for Super-Resolution Semantic Segmentation of Urban Scene Images

**DOI:** 10.3390/s24144522

**Published:** 2024-07-12

**Authors:** Ruijun Shu, Shengjie Zhao

**Affiliations:** 1 College of Electronic and Information Engineering, Tongji University, Shanghai 201804, China; ruijun_shu@tongji.edu.cn; 2 Shanghai Institute of Microsystem and Information Technology, Chinese Academy of Sciences, Shanghai 200050, China; 3 School of Software Engineering, Tongji University, Shanghai 201804, China

**Keywords:** image semantic segmentation, super-resolution semantic segmentation, multi-resolution learning, semantic edge enhancement

## Abstract

Super-resolution semantic segmentation (SRSS) is a technique that aims to obtain high-resolution semantic segmentation results based on resolution-reduced input images. SRSS can significantly reduce computational cost and enable efficient, high-resolution semantic segmentation on mobile devices with limited resources. Some of the existing methods require modifications of the original semantic segmentation network structure or add additional and complicated processing modules, which limits the flexibility of actual deployment. Furthermore, the lack of detailed information in the low-resolution input image renders existing methods susceptible to misdetection at the semantic edges. To address the above problems, we propose a simple but effective framework called multi-resolution learning and semantic edge enhancement-based super-resolution semantic segmentation (MS-SRSS) which can be applied to any existing encoder-decoder based semantic segmentation network. Specifically, a multi-resolution learning mechanism (MRL) is proposed that enables the feature encoder of the semantic segmentation network to improve its feature extraction ability. Furthermore, we introduce a semantic edge enhancement loss (SEE) to alleviate the false detection at the semantic edges. We conduct extensive experiments on the three challenging benchmarks, Cityscapes, Pascal Context, and Pascal VOC 2012, to verify the effectiveness of our proposed MS-SRSS method. The experimental results show that, compared with the existing methods, our method can obtain the new state-of-the-art semantic segmentation performance.

## 1. Introduction

Semantic segmentation of urban scene images plays an important role in many real-life applications, such as autonomous driving, robot sensing, etc. Deep learning technology greatly promotes the performance of semantic segmentation. New methods and models are emerging, e.g., Ref. [1] proposes a novel soft mining contextual information beyond-image paradigm named MCIBI++ to boost the pixel-level representations; Ref. [2] proposes a Residual Spatial Fusion Network (RSFNet) for RGB-T semantic segmentation; Ref. [3] develops the Knowledge Distillation Bridge (KDB) framework for few-sample unsupervised semantic segmentation; and Ref. [4] proposes a pixel-wise contrastive algorithm, dubbed as PiCo, for semantic segmentation in the fully supervised learning setting. However, in applications such as autonomous driving, where the safety response time is less than 0.1 s, the high computational cost required by the deep learning model limits the deployment of the semantic segmentation model on mobile devices with limited resources.

There have been some compression algorithms, such as pruning [5,6], knowledge distillation [7,8], and quantification [9,10], or compact modules design [11,12,13,14], to lower the computational cost. The above methods mainly alleviate the problem of high computing cost through network parameter compression. Intuitively, the high resolution of the input image is also a critical reason for the high computational cost. Therefore, super-resolution semantic segmentation (SRSS) is an alternative method to reduce the computational overhead. It reduces the resolution of the original input image as the input of a given semantic segmentation network and up-samples the generated low-resolution semantic prediction results to obtain the same resolution as the original input image. The current challenges facing the super-resolution semantic segmentation task mainly include: (1) down-sampling high-resolution images will lose much detailed information, especially for small objects, which results in failing to keep impressive pixel-wise semantic prediction performance; and (2) the design of adding complex computational modules or up-sampling modules drastically increases the complexity of model inference, which goes against the original intention of keeping lower computational cost. Thus, in this paper, we will propose a novel SRSS method which can simultaneously obtain impressive high-resolution segmentation results and maintain a lower computational cost.

In recent years, there are a few scholars who have begun to study this issue [15,16,17,18]. Zhang et al. [15] proposed an HRFR method based on knowledge distillation. The HRFR used a SRR module to up-sample the extracted features learned in the super-resolution network to recover high-resolution features and decoded them accordingly to obtain high-resolution semantic segmentation results. HRFR first tried to solve the SRSS problem, but it needed to add new processing modules and there was still a lot of room for performance improvement. Wang et al. [16] proposed a dual super-resolution learning method called DSRL, which was able to keep high-resolution representations with low-resolution input by utilizing super-resolution and feature affine. DSRL adopts an image reconstruction task to guide the training of the super-resolution semantic segmentation task, but the two tasks are not intrinsically related, and thus, the guidance for training is of limited effectiveness. While DSRL does not change the parsing network structure, the performance degradation is significant. Jiang et al. [17] subsequently proposed a relation calibrating network (RCNet) to restore the high-resolution representations by capturing the relation’s propagation from low resolution to high resolution and using the relation map to calibrate the feature information. RCNet designs an extra high-complexity module to achieve a boost in performance, but increases inference complexity, and there is room for further performance improvement. Although the RCNet achieves the state-of-the-art prediction performance, it requires modifications to the given semantic segmentation network structure, which limits the flexibility in practical applications. Liu et al. [18] proposed a super-resolution semantic segmentation method for remote sensing images in which the super-resolution guided branch is designed to supplement rich structure information and guide the semantic segmentation. Although the method [18] has been proven to be effective in remote sensing datasets, the effectiveness of the method in the urban scene needs to be further evaluated, as the urban scene images are more complex than remote sensing images. In addition, the existing methods overlook the focus on semantic edges, where false positives always exist due to the loss of some detailed information in reduced-resolution input images.

To solve the above dilemma, we propose a novel method, MS-SRSS, which is a simple but effective framework that does not change the original structure of the given semantic segmentation network and does not increase computational costs. Specifically, a multi-resolution learning mechanism (MRL) is proposed for capturing multi-resolution representations and promoting the representation ability of the model. In the training process, we construct a dual branch structure, with one branch for reduced-resolution image input and the other for high-resolution image input. The multi-resolution representations can be captured by the shared encoder weights during the joint training of two branches. In addition, in order to further improve the segmentation accuracy on semantic boundaries, a semantic edge enhancement (SEE) loss is proposed to impose soft constraints on semantic edges through local structural information. After the training process is accomplished, we only utilize a super-resolution branch with simple up-sampling operations on the low-resolution segmentation results to obtain high-resolution semantic segmentation results in the inference stage. In summary, our main contributions include:

1. We propose a novel super-resolution semantic segmentation method called MS-SRSS, which can achieve improved semantic segmentation results without modifying the structure of the original given semantic segmentation network.
2. We propose a multi-resolution learning mechanism (MRL) and a semantic edge enhancement loss (SEE) to boost the representation ability of the model and alleviate the problem of semantic edge confusion due to the low resolution of the input image.
3. Extensive experiments demonstrate that our proposed method outperforms the baseline method and several state-of-the-art super-resolution semantic segmentation methods with different image input resolutions.

## 2. Methodology

In this section, we first introduce an overview of our proposed framework for the super-resolution semantic segmentation (SRSS) in Section 2.1. Then, we give a specific description of the two main strategies of our proposed method, including the multi-resolution learning mechanism (MRL) and the semantic edge enhancement loss (SEE), explained in Section 2.2 and Section 2.3, respectively. Finally, we present the optimization objective function briefly.

### 2.1. Overview

The goal of the SRSS task is to take the down-sampled raw image as the input of the semantic segmentation network and output the semantic segmentation prediction results with the same resolution as the raw image. In this way, the inference process can save a lot of computational cost, which makes an important contribution to the application of semantic segmentation in real scenarios.

In this paper, we propose a multi-resolution learning- and semantic edge enhancement-based super-resolution semantic segmentation method (MS-SRSS) for the super-resolution semantic segmentation task. Specifically, to alleviate the problem of missing information due to low-resolution input images, we present a novel multi-resolution learning mechanism (MRL) which aims to motivate the semantic segmentation network to capture multi-resolution representational information for more precise semantic segmentation results. In addition, low-resolution input images increase the difficulty of semantic segmentation at object edges, especially for small objects or nearby objects with similar appearance. Therefore, we further propose a semantic edge enhancement loss (SEE) function and integrate it into the overall loss objective function to motivate the network to focus on the edge region. Our proposed framework can directly use existing encoder–decoder-based networks (e.g., Deelabv3+, PSPNet, OCNet, etc.) to generate semantic segmentation results without changing the network structure or adding additional modules. The encoder–decoder structure is a classic and effective network structure for semantic segmentation. During the training stage, the two branches are trained in the fashion of multi-task learning, while in the inference stage, it is the super-resolution branch that actually performs the super-resolution semantic segmentation inference.

**Figure 1:**
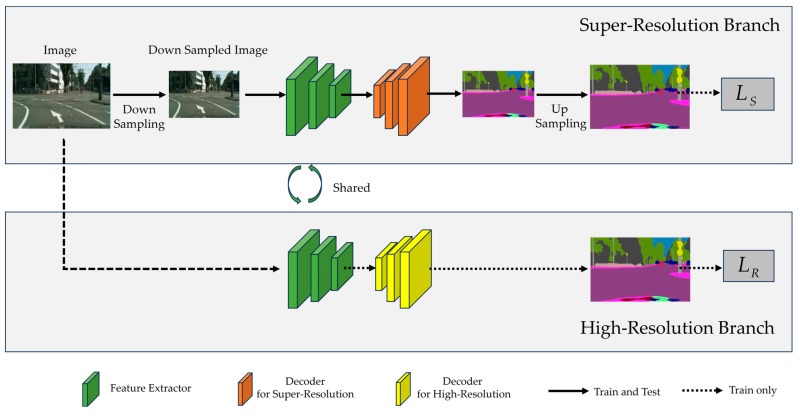
The overview of our proposed framework, containing a high-resolution branch and a super-resolution branch, in which the structure of the semantic segmentation network is the same and the encoder weights are shared. The two branches are trained jointly, and the training loss consists of $L_{S}$ and $L_{R}$ for the super-resolution branch and the high-resolution branch, respectively. In the inference phase, only the super-resolution branch is utilized.

Figure 1 provides an overview of the proposed framework. Assuming that a raw RGB image is of size *H* × *W* × 3, the input image to the super-resolution branch is the low-resolution image of size (*H*/*K*) × (*W*/*K*) × 3 obtained by reducing the raw image resolution through a down-sampling operation. Here, *H* and *W* refer to the raw image height and width, respectively, while *K* is the down-sampling coefficient. After the low-resolution semantic segmentation results are obtained by the semantic segmentation network, the high-resolution semantic prediction results are obtained by a simple up-sampling operation. We choose the simple bilinear method as the up-sampling operation. Our framework aims to obtain precise super-resolution segmentation results of size *H* × *W* × *C*, where *C* represents the number of semantic categories.

### 2.2. Multi-Resolution Learning Mechanism

The core of the MRL mechanism is the introduction of a high-resolution semantic segmentation prediction branch to support the training process. Our motivations for adopting multi-resolution learning include: (1) the high-resolution images in the dataset contain richer color and texture information and clearer information about the boundaries of objects, especially small objects, and the multi-resolution learning mechanism can make full use of the above information; (2) the multi-resolution learning can improve the feature representation ability of the network by learning different resolution representations at the same time, ultimately improving the semantic segmentation performance.

As shown in Figure 1, our framework contains two branches during training, namely, the high-resolution branch and the super-resolution branch. Among them, the super-resolution branch is the main task, and the high-resolution branch is the auxiliary task. These two branches use the same semantic segmentation network, where the encoder weights are shared and the decoder weights are branch-specific. To put it another way, the high-resolution branch and the super-resolution branch use the same encoder, but different decoders. For the super-resolution branch, the raw image is firstly reduced by a down-sampling operation and then fed into the given semantic segmentation network to generate low-resolution semantic probability maps, which are further used to obtain the final high-resolution semantic segmentation results through the up-sampling operation. For the up-sampling operation, we choose the simple bilinear method. In the high-resolution branch, the input is the raw image, and the sizes of the generated semantic segmentation results are consistent with the raw images.

During the training stage, the two branches are trained in the fashion of multi-task learning. For each training iteration of multi-resolution learning, we feed the high-resolution image and its corresponding low-resolution image into the semantic segmentation network in succession for learning, and this learning method integrates the advantages of contrastive learning and multi-task learning so that the feature encoder of the semantic segmentation network is able to extract the affinity features applicable to both the high-resolution and the low-resolution images, thus improving the super-resolution semantic segmentation performance. Losses for both branches are added together with a trade-off coefficient, and gradients are taken for the collection of all parameters. The MRL described above is similar to the anti-forgotten configuration, which causes the feature extractor of the semantic segmentation network to acquire different resolution representations simultaneously, boosting the representational capacity and semantic segmentation performance.

### 2.3. Semantic Edge Enhancement Loss

The Structure Similarity Index Measure (SSIM) [19] implements constraints through the similarity of the structural information of images, which, in addition to its important role in assessing image quality [20], also shows potential in optimizing saliency map prediction [21], rate control [22], rate-distortion optimization [23], object detection [24], image retrieval [25], and hyperspectral band selection [26]. We further extend SSIM by applying this structural information constraint to multi-class semantic segmentation and propose a semantic edge enhancement (SEE) loss.

Specifically, patches with a size of *M × M* are cropped from each predicted semantic category probability map and its corresponding truth map. And the SEE loss is defined as:

(1) $$L_{SEE}=\sum_{i=1}^{C}\sum_{j=1}^{U}\left\{1-\frac{\left(2\mu_{i,j}^{x}\mu_{i,j}^{y}+\varepsilon_{1}\right)\left(2\delta_{i,j}^{x}\delta_{i,j}^{y}+\varepsilon_{2}\right)}{\left(\mu_{i,j}^{x}+\mu_{i,j}^{y}+\varepsilon_{1}\right)\left(\delta_{i,j}^{x}+\delta_{i,j}^{y}+\varepsilon_{2}\right)}\right\}$$

where *C* denotes the number of semantic categories; *U* denotes the number of patches; $\mu_{i,j}^{x}$, $\mu_{i,j}^{y}$ are the mean value of the *j*-th patch in the *i*-th semantic segmentation probability map and the corresponding groundtruth; $\delta_{i,j}^{x}$, $\delta_{i,j}^{y}$ are the standard deviations of the *j*-th patch in the *i*-th semantic segmentation probability and the corresponding groundtruth; and $\varepsilon_{1}$ and $\varepsilon_{2}$ are constants to avoid dividing by zero.

We further elaborate on how SEE loss improves edge accuracy. When the *j*-th patch is assigned to the *i*-th semantic category, the mean value $\mu_{i,j}^{x}$ is observed to be larger, and the standard deviation $\delta_{i,j}^{x}$ is found to be smaller. Conversely, when the *j*-th patch is at the semantic edge, the $\mu_{i,j}^{x}$ is smaller and the $\delta_{i,j}^{x}$ is larger. Therefore, we implement constraints on semantic edges by narrowing the gap between the predicted mean values and the standard deviation of each image patch and their corresponding groundtruth values. This soft supervision based on the structural statistical information within the image patches provides better generalization than the hard supervision that applies L1 or L2 loss to the pixels at the semantic edges.

### 2.4. Optimization

As shown in Figure 1, the whole objective function *L* of our training framework is composed of two parts, the loss of the super-resolution branch $L_{S}$ and the loss of the high-resolution branch $L_{R}$:

(2) $$L=L_{S}+\lambda_{R}L_{R}$$

where $\lambda_{R}\geq 0$ is trade-off coefficient for controlling the influence of the high-resolution branch. 

Specifically, for the super-resolution branch, we use the multi-class cross-entropy loss (CE) and the semantic edge-enhanced loss (SEE) to guide the training process simultaneously:

(3) $$L_{S}=L_{CE}^{S}+\lambda_{SEE}L_{SEE}^{S}$$

where $\lambda_{SEE}\geq 0$ is the coefficient to balance the different loss terms. $L_{CE}^{S}$ is the SEE loss term (refer to Equation (1)). $L_{CE}^{S}$ is the multi-class cross-entropy loss for the super-resolution branch, which can be presented as:

(4) $$L_{CE}^{S}=-\frac{1}{N}\sum_{i=1}^{N}y_{i}log\left(p_{i}^{s}\right)$$

where $p_{i}^{s}$ and $y_{i}$ refer the predicted semantic probability and the corresponding category for pixel *i*, and *N* means the total number of pixels in the raw image.

For the high-resolution branch, we only use the multi-class cross-entropy loss $L_{CE}^{R}$ to guide the training:

(5) $$L_{R}=L_{CE}^{R}=-\frac{1}{N}\sum_{i=1}^{N}y_{i}log\left(p_{i}^{r}\right)$$

where $p_{i}^{r}$ and $y_{i}$ refer the predicted semantic probability and the corresponding category for pixel *i*, and *N* means the total number of pixels in the raw image.

In summary, the overall optimization objective can be expressed as follows:

(6) $$L=L_{CE}^{S}+\lambda_{SEE}L_{SEE}^{S}+\lambda_{R}L_{CE}^{R}$$

In our experiments, we set both the trade-off parameters $\lambda_{R}$ and $\lambda_{SEE}$ to 1.

## 3. Results

### 3.1. Datasets

1. Cityscapes: The Cityscapes dataset [27] is a classical and commonly used dataset for urban street scene understanding which is captured across 50 cities in different seasons. It contains 2975 training, 500 validation, and 1525 test images with fine-grained annotations. The resolution of each image in the dataset is 1024 × 2048. The dataset contains 19 semantic categories. We chose Cityscapes as our primary benchmark and evaluated the performance on the validation set over the 19 semantic classes.
2. Pascal Context: The Pascal Context dataset [28] is a challenging benchmark which contains both indoor and outdoor scenarios. It has a total of 10,103 images, among which 4998 images are used as a training set and the other 5105 images are used as a validation set. The dataset has a total of 459 categories. Following [17], 59 object categories that appear most frequently are used as semantic labels, and the other categories are all set as the background class. We evaluate the performance on the validation and test set over these 59 classes and one background class.
3. Pascal VOC 2012: The PASCAL VOC 2012 dataset [29] is one of the standard benchmarks for semantic segmentation tasks. Our experiments use the augmented annotation set consisting of 10,582, 1449, and 1456 images in the training set, validation set, and test set, respectively. The dataset involves 20 foreground object classes and one background class. We evaluate the performance on the validation set.

We demonstrate the effectiveness and efficiency of our proposed super-resolution semantic segmentation method based on the above three datasets. In each dataset, training images are used for model training, and then comparison experiments are performed utilizing the calibration images.

### 3.2. Implementation Details

#### 3.2.1. Semantic Segmentation Networks

To verify the effectiveness and generalization of our proposed framework, we conducted ablation experiments and comparison experiments using several classic and effective semantic segmentation networks, namely, Deeplabv3+ [30], PSPNet [31], and OCNet [32]. These networks are encoder–decoder-based networks, which are commonly used in SRSS tasks. In addition, we employ ResNet-50 as the backbone, and the output strides are set to 8 for PSPNet and OCNet and 16 for Deeplabv3+.

#### 3.2.2. Training Setup

The experiments were implemented on the PyTorch platform. We conducted ablation studies and comparison experiments on the Cityscapes, Pascal Context, and Pascal VOC 2012 datasets.

For the Cityscapes dataset, our network was trained with mini-batch stochastic gradient descent (SGD), where the momentum was set to 0.9 and the weight decay was set to 0.0001. We adopted a poly learning rate policy as the learning rate scheduler, where the initial learning rate was set to 0.01 and decayed after each iteration with a power of 0.9. In addition, we applied random cropping and random left–right flipping as the data augmentation techniques during the training process. In our experiments, we set the down-sampling coefficient *K* to control the resolution reduction degree of the raw image. The down-sampling coefficients included 2, 4, and 8. For example, when the down-sampling coefficient was set to 2, the resolution of the input image was down-sampled from 1024 × 2048 to 512 × 1024. When compared with various down-sampling coefficients, the crop sizes were set to 384 × 384, 192 × 192, and 96 × 96 for down-sampling coefficients 2, 4, and 8, respectively. The batch size was set to 8. We trained our proposed models for 180 epochs. 

For the Pascal Context dataset and the Pascal VOC 2012 dataset, we set the initial learning rate to 0.001. When compared with various down-sampling coefficients, the crop sizes were set to 192 × 192, 96 × 96, and 48 × 48 for down-sampling coefficients 2, 4, and 8, respectively. The batch size was set to 16. The other training settings were the same as those for the Cityscapes dataset.

### 3.3. Evaluation Metrics

In our experiments, we used the commonly adopted evaluation metric—mean intersection over union (mIoU)—to evaluate the semantic segmentation performance of the proposed method and the floating point of operations (FLOPs) to evaluate the computational cost during the interference stage. Specifically, the mIoU was obtained by calculating the IoU of each semantic class and then calculating the average over these classes.

### 3.4. Ablation Study

#### 3.4.1. Effect of Algorithmic Components

We first investigated the effectiveness of the MRL mechanism and the SEE loss in our proposed method using Deeplabv3+ with ResNet50 on the Cityscapes dataset, where the down-sampling coefficient was set to 2 and the output resolution was 1024 × 2048. For the baseline method, the semantic segmentation model was trained directly using down sampled images, high-resolution labels, and the bilinear as the up-sampling operator. Table 1 shows that the SEE loss and the MRL mechanism improved the mIoU by 2.60% and 3.02% compared to the baseline method, respectively. In addition, the introduction of SEE loss could further improve the semantic segmentation performance by further optimizing semantic edges. Finally, our method improved the mIoU by 3.20% compared to the baseline method.

**Figure 2:**
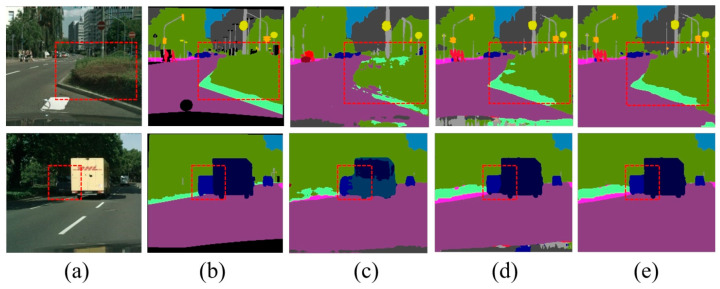
Qualitative experimental comparison results. The resolution of input image is 512 × 1024 and resolution of the output semantic results is 1024 × 2048. The figure above displays the local results for easier viewing and comparison. (**a**) Input image, (**b**) groundtruth image, (**c**) the baseline results, (**d**) ours (+MRL) results, and (**e**) ours (+MRL +SEE) results. The red dashed squares point out the location of the comparison.

We also present some semantic segmentation results in Figure 2, which shows the qualitative comparison results when the input image resolution was reduced to 512 × 1024. It can be seen that the MRL mechanism improved the segmentation results in the shrub and truck regions, while the correction of the semantic edge regions was further achieved by further introducing the SEE loss.

In order to validate the generalization of proposed method, we further carried out similar experiments on the Pascal Context dataset. Compared to the baseline method, Table 2 shows that the SEE loss and the MRL mechanism improved the mIoU by 0.92% and 1.20%, respectively. In addition, the MRL mechanism combined with SEE loss could further improve the mIoU by 1.57% compared to the baseline method.

#### 3.4.2. Comparison with Other Up-Sampling Methods

In order to verify that adopting the bilinear as the up-sampling operator in our method is reasonable, we further compared our approach with other up-sampling operators using Deeplabv3+ with ResNet50 on the Cityscapes dataset, where the down-sampling coefficient was set to 2 and the output resolution was 1024 × 2048. The terms “Bilinear + conv” and “Nearest + conv” indicate the addition of an extra 3 × 3 convolution layer after bilinear and nearest, respectively. “Deconv” [33], “Pixelshuffle” [34], and “CARAFE” [35] refer to popular methods for up-sampling with learnable parameters. Table 3 shows the comparison results, indicating that the extra convolution layer and other learnable up-sampling methods had a limited effect on improving semantic segmentation precision, but increased the inference computational cost. Based on the results of the above comparation experiments, it is reasonable to choose the bilinear as the up-sampling operator in our method.

#### 3.4.3. Effects of $\lambda_{R}$ and $\lambda_{SEE}$

We conducted the ablation study of $\lambda_{R}$ and $\lambda_{SEE}$ in our proposed method using Deeplabv3+ with ResNet50 on the Cityscapes dataset, where the down-sampling coefficient was set to 2 and the output resolution was 1024 × 2048. 

$\lambda_{R}$ is the trade-off coefficient for controlling the influence of the high-resolution branch on the super-resolution branch. The setting of the parameter $\lambda_{R}$ has some impact on the results of super-resolution semantic segmentation branch. In the ablation study for $\lambda_{R}$, we set $\lambda_{R}$ to 0.0, 0.5, 1.0, 1.5, and 2.0, respectively, while the $\lambda_{SEE}$ was fixed to 0.0. The ablation study results for $\lambda_{R}$ are shown in Table 4. The experimental results show that choosing too small a value led to insufficient influence, and choosing too large a value led to too much influence. In subsequent comparison experiments, we set $\lambda_{R}$ to 1.0 based on the ablation study results.

$\lambda_{SEE}$ is the coefficient to balance the CE loss and SEE loss in the super-resolution branch. In the ablation study for $\lambda_{SEE}$, we set $\lambda_{SEE}$ to 0.0, 0.5, 1.0, 1.5, and 2.0, respectively, while the $\lambda_{R}$ was fixed at 0.0. The ablation study results for $\lambda_{SEE}$ are shown in Table 5. The experimental results show that when the $\lambda_{SEE}$ took a value greater than 1.0, the performance did not continue to increase. In subsequent comparison experiments, we set $\lambda_{SEE}$ to 1.0 based on the ablation study results.

### 3.5. Results on Cityscapes

To demonstrate the effectiveness, efficiency, and generality of our proposed MS-SRSS, we compared it with the state-of-the-art super-resolution semantic segmentation method RCNet [13] on the Cityscapes dataset.

On the one hand, in order to verify that the proposed MS-SRSS would be effective for different down-sampling coefficients, we first carried out the comparison experiments on the Cityscapes dataset for different down-sampling coefficients. We adopted the Deeplabv3+ with ResNet50 as the semantic segmentation network, where the output stride was set to 16. The output sizes were set to 1024 × 2048 for all the experiments. The image segmentation accuracies and computational cost corresponding to different down-sampling coefficients are shown in Table 6. The results show that, compared with the SOTA method RCNet, our proposed MS-SRSS improved mIoU at all down-sampling coefficients while using only about 70% of the inference computation. Specifically, MS-SRSS improved the mIoU from 74.22%, 66.06%, and 51.03% to 75.22%, 66.26%, and 53.25% at down-sampling coefficients of 2, 4, and 8, respectively. Overall, the mIoU improved by about 1.14% on average. In summary, the experimental results demonstrate that the proposed method is effective for different down-sampling coefficients. 

**Figure 3:**
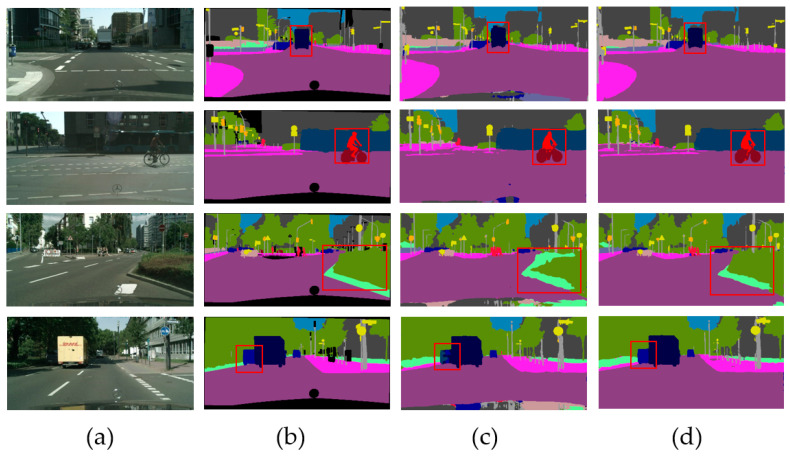
Qualitative experimental comparison results of RCNet and our proposed MS-SRSS on Cityscapes validation set. The down-sampling coefficient was set to 2. The figure displays the local results for easier viewing and comparison. (**a**) Input image, (**b**) groundtruth image, (**c**) RCNet results, and (**d**) our MS-SRSS results. Compared with the RCNet, the semantic prediction results improved with our proposed MS-SRSS. The red squares point out the location of the comparison.

In order to show the effect of comparison experiments more intuitively, we also present some qualitative semantic segmentation results on the Cityscapes validation set in Figure 3. It shows the comparison results of the RCNet and our proposed MS-SRSS at the down-sampling coefficient 2. It can be seen that our MS-SRSS, which is based on the MRL mechanism and the SEE loss, improved the segmentation results in the shrub, truck, and rider regions.

On the other hand, in order to verify that our proposed MS-SRSS was robust to different semantic segmentation networks, we further carried out the comparison experiments with RCNet on the Cityscapes dataset using PSPNet, OCNet, and Deeplabv3+. The ResNet50 was employed as the backbone for all networks, and the output strides were set to 8 for PSPNet and OCNet and 16 for Deeplabv3+. The down-sampling coefficient was fixed at 2, and the output sizes were set to 1024 × 2048 for all the experiments. The image segmentation accuracies and computational costs corresponding to different networks are shown in Table 7. The results show that, compared with the RCNet method, our proposed MS-SRSS method improved the mIoU for all networks in the experiments. Specifically, our method improved the mIoU by 1.20%, 0.73%, and 1.00% for PSPNet, OCNet, and Deeplabv3+, respectively. Overall, the mIoU improved by about 0.98% on average while using only about 84.96% of the inference computation. In addition, the overall Acc and Mean Acc of our proposed MS-SRSS achieved 95.54% and 83.16%. In summary, the experimental results demonstrate that our method is robust for various semantic segmentation networks.

To demonstrate the advantages of the proposed MS-SRSS in terms of semantic segmentation accuracy and inference computational cost, we further compared it with the DSRL method [12], which uses the deeper ResNet-101 as the backbone network. Our comparison experiments were carried out on the Cityscapes dataset using Deeplabv3+ as the semantic segmentation network. The comparison results are shown in Table 8. Compared to the DSRL, our method MS-SRSS improved the mIoU by 3.22% while using only about 79.74% of the inference computation cost and 68.00% of the model size. These results can consistently demonstrate the effectiveness and low inference cost of the proposed MS-SRSS.

### 3.6. Results on Pascal Context

To further demonstrate the effectiveness and generality of the proposed MS-SRSS on various datasets, we also compared it with the SOTA method RCNet [13] on the Pascal Context dataset, which is a more challenging dataset containing images of various resolutions. We adopt Deeplabv3+ with ResNet50 as the semantic segmentation network, where the output stride was set to 16. The output resolutions were set to the same sizes as the groundtruth. The input images were down-sampled based on different down-sampling coefficients. Table 9 displays the experimental results, indicating that the proposed MS-SRSS resulted in an improvement in mIoU for different down-sampling coefficients. Specifically, our method improved the mIoU by 1.54%, 0.55%, and 0.61% compared to the RCNet for down-sampling coefficients of 2, 4, and 8, respectively. Overall, the mIoU was improved by about 0.90% on average. In summary, the experimental results show that the MS-SRSS can achieve improved semantic segmentation performance compared to the SOTA method RCNet for various down-sampling coefficients.

### 3.7. Results on Pascal VOC 2012

In addition, we conducted comparation experiments on the Pascal VOC 2012 dataset. The comparation experiments with the proposed MS-SRSS and the baseline using the bilinear up-sampling operation were based on Deeplabv3+, where the ResNet50 was employed as the backbone and the output stride was set to 16. The baseline means that the semantic segmentation model was directly trained using low-resolution images and high-resolution labels for end-to-end direct supervision. The input images were down-sampled based on different down-sampling coefficients, and the output resolutions were set to the same sizes as the groundtruth. Table 10 presents the experimental results, indicating that the proposed MS-SRSS improved the mIoU for different down-sampling coefficients. Specifically, our method improved the mIoU by 1.88%, 1.34%, and 2.06% compared to the baseline for down-sampling coefficients of 2, 4, and 8, respectively. Overall, the mIoU was improved by about 1.76% on average.

## 4. Discussion

Our work mainly focused on the task of super-resolution semantic segmentation. Compared with previous works, we made some progress in the improvement of semantic segmentation performance, but there is still room for performance improvement, and further efforts are needed. In addition, this work can be further extended to other tasks, such as object detection and pose estimation. 

## 5. Conclusions

In this paper, we propose a novel super-resolution semantic segmentation (SRSS) method called MS-SRSS. By using the multi-resolution learning (MRL) mechanism and the semantic edge enhancement (SSE) loss function, our proposed MS-SRSS method can effectively improve the semantic prediction accuracy for SRSS without increasing the extra computing overhead. Specifically, the MRL mechanism is presented to motivate the network to acquire multi-resolution semantic representation information and improve the semantic segmentation precision without changing the original network structure. The semantic edge enhancement (SEE) loss is introduced to further improve the segmentation precision at semantic edges. 

To evaluate the specific contributions of MRL and SEE to the proposed method, we performed targeted ablation experiments. The experimental results show the important role of both components in our method and their contributions to the improvement of performance. To further validate the effectiveness and efficiency of our method, we conducted extensive comparison experiments on the Cityscapes, Pascal Context, and Pascal VOC 2012 datasets, which are representative benchmarks in the field of urban scene perception. The experimental results also show that our method achieved better mIoU than the state-of-the-art super-resolution semantic segmentation methods at different down-sampling coefficients with lower FLOPs.

## Figures and Tables

**Table 1:** Experiments on Cityscapes validation set using Deeplabv3+ with ResNet50. The output resolution is 1024 × 2048.

Method	Down-Sampling Coefficient	mIoU (%)
Baseline	2	72.02
+SEE	2	73.89
+MRL	2	75.04
+MRL +SEE	2	75.22

**Table 2:** Experiments on Pascal Context validation set using Deeplabv3+ with ResNet50. The output resolutions are set to the same size as the groundtruth.

Method	Down-Sampling Coefficient	mIoU (%)
Baseline	2	42.57
+SEE	2	42.96
+MRL	2	43.77
+MRL +SEE	2	44.14

**Table 3:** Comparison with other up-sampling methods on Cityscapes validation set using Deeplabv3+ with ResNet50. The input sizes and the output sizes of the experiments are set to 512 × 1024 and 1024 × 2048, respectively.

Method	mIoU (%)	GFLOPs
Nearest	71.22	141.76
Nearest + conv	71.76	148.64
Bilinear + conv	72.61	148.64
Deconv	72.20	165.99
Pixelshuffle	72.26	148.61
CARAFE	72.72	147.64
Bicubic	72.18	141.76
Ours (Bilinear)	72.02	141.76

**Table 4:** Ablation results for $\lambda_{R}$ on Cityscapes validation set using Deeplabv3+ with ResNet50. The input sizes and the output sizes of the experiments were set to 512 × 1024 and 1024 × 2048, respectively.

$\lambda_{R}$	0.0	0.5	1.0	1.5	2.0
**mIoU (%)**	72.02	74.67	75.04	74.53	74.12

**Table 5:** Ablation results for $\lambda_{SEE}$ on Cityscapes validation set using Deeplabv3+ with ResNet50. The input sizes and the output sizes of the experiments are set to 512 × 1024 and 1024 × 2048, respectively.

$\lambda_{SEE}$	0.0	0.5	1.0	1.5	2.0
**mIoU (%)**	72.02	72.87	73.89	73.76	73.85

**Table 6:** Experiments of our proposed MS-SRSS compared to the RCNet on Cityscapes validation set using Deeplabv3+ with ResNet50. The output sizes of the experiments are set to 1024 × 2048.

Method	Down-Sampling Coefficients	mIoU (%)	GFLOPs
RCNet	2	74.22	202.33
	4	66.06	49.33
	8	51.03	12.33
MS-SRSS	2	75.22	141.76
	4	66.26	35.44
	8	53.25	8.86

**Table 7:** Experiments of our proposed method MS-SRSS compared to the RCNet on Cityscapes validation set. The down-sampling coefficient was fixed at 2, and the output sizes of the experiments were set to 1024 × 2048.

Method	Network	mIoU (%)	GFLOPs
RCNet	PSPNet	72.73	370.45
	OCNet	72.18	340.03
	Deeplabv3+	74.22	202.33
Liu et al. [18]	PSPNet	72.52	354.79
	OCNet	72.03	324.82
	Deeplabv3+	73.87	138.84
MS-SRSS	PSPNet	73.93	354.79
	OCNet	72.91	324.82
	Deeplabv3+	75.22	138.84

**Table 8:** Experiments compared to DSRL on Cityscapes validation set. The output sizes of the experiments were set to 1024 × 2048.

Method	Model Size (M)	Down-Sampling Coefficient	mIoU (%)	GFLOPs
DSRL (Deeplabv3+ with ResNet101)	59.34	2	72.00	177.77
MS-SRSS (Deeplabv3+ with ResNet50)	40.35	2	75.22	141.76

**Table 9:** Experiments on various input resolutions on Pascal Context validation set using Deeplabv3+ with ResNet50. The output sizes of the experiments were set to the same sizes as the groundtruth.

Method	Down-Sampling Coefficients	mIoU (%)
RCNet	2	43.37
	4	34.24
	8	21.36
MS-SRSS	2	44.91
	4	34.79
	8	21.97

**Table 10:** Experiments on various input resolutions on Pascal VOC 2012 validation set using Deeplabv3+ with ResNet50. The output sizes of the experiments were set to the same sizes as the groundtruth.

Method	Down-Sampling Coefficients	mIoU (%)
Baseline	2	61.89
	4	44.59
	8	24.59
MS-SRSS	2	63.77
	4	45.93
	8	26.65

## Data Availability

Data are contained within the article.
